# Machine learning assisted reflectance spectral characterisation of coronary thrombi correlates with microvascular injury in patients with ST-segment elevation acute coronary syndrome

**DOI:** 10.3389/fcvm.2022.930015

**Published:** 2022-09-20

**Authors:** Rafail A. Kotronias, Kirsty Fielding, Charlotte Greenhalgh, Regent Lee, Mohammad Alkhalil, Federico Marin, Maria Emfietzoglou, Adrian P. Banning, Claire Vallance, Keith M. Channon, Giovanni Luigi De Maria

**Affiliations:** ^1^Oxford Heart Centre, National Institute for Health and Care Research (NIHR) Biomedical Research Centre, Oxford University Hospitals, Oxford, United Kingdom; ^2^Department of Cardiovascular Medicine, University of Oxford, Oxford, United Kingdom; ^3^Department of Chemistry, University of Oxford, Oxford, United Kingdom; ^4^Nuffield Department of Surgical Sciences, University of Oxford, Oxford, United Kingdom; ^5^Cardiothoracic Centre, Freeman Hospital, Newcastle, Translational and Clinical Research Institute, Newcastle University, Newcastle upon Tyne, United Kingdom

**Keywords:** coronary thrombus, STEACS, reflectance spectroscopy, machine learning, coronary microvascular injury, coronary microvascular dysfunction (CMD)

## Abstract

**Aims:**

We set out to further develop reflectance spectroscopy for the characterisation and quantification of coronary thrombi. Additionally, we explore the potential of our approach for use as a risk stratification tool by exploring the relation of reflectance spectra to indices of coronary microvascular injury.

**Methods and results:**

We performed hyperspectral imaging of coronary thrombi aspirated from 306 patients presenting with ST-segment elevation acute coronary syndrome (STEACS). Spatially resolved reflected light spectra were analysed using unsupervised machine learning approaches. Invasive [index of coronary microvascular resistance (IMR)] and non-invasive [microvascular obstruction (MVO) at cardiac magnetic resonance imaging] indices of coronary microvascular injury were measured in a sub-cohort of 36 patients. The derived spectral signatures of coronary thrombi were correlated with both invasive and non-invasive indices of coronary microvascular injury. Successful machine-learning-based classification of the various thrombus image components, including differentiation between blood and thrombus, was achieved when classifying the pixel spectra into 11 groups. Fitting of the spectra to basis spectra recorded for separated blood components confirmed excellent correlation with visually inspected thrombi. In the 36 patients who underwent successful thrombectomy, spectral signatures were found to correlate well with the index of microcirculatory resistance and microvascular obstruction; *R*^2^: 0.80, *p* < 0.0001, *n* = 21 and *R*^2^: 0.64, *p* = 0.02, *n* = 17, respectively.

**Conclusion:**

Machine learning assisted reflectance spectral analysis can provide a measure of thrombus composition and evaluate coronary microvascular injury in patients with STEACS. Future work will further validate its deployment as a point-of-care diagnostic and risk stratification tool for STEACS care.

## Introduction

ST-segment elevation acute coronary syndrome (STEACS) presentation is secondary to atherosclerotic plaque disruption (erosion or rupture) leading to a prothrombotic milieu with subsequent thrombotic occlusion of the culprit artery and consequent myocardial necrosis. The introduction of primary percutaneous coronary intervention (pPCI) services, has led to significant reductions in mortality following a STEACS (1). Despite prompt coronary blood flow restoration, subsequent cardiac failure is on the rise due to a variety of pathological mechanisms culminating into suboptimal downstream myocardium perfusion (1, 2). Coronary microvascular injury is a key mechanism of prognostic importance that is predominantly, yet not exclusively, related to atherothrombotic material embolisation following mechanical flow restoration (2–5).

Coronary thrombus aspiration with manual thrombectomy can be used in patients with high thrombus burden (6), with a patient level meta-analysis of randomised studies identifying a trend towards better clinical outcomes (7). Beyond its potential therapeutic role, thrombus retrieval may prove useful for stratified medicine approaches in STEACS care. Indeed, erythrocyte-rich, macroscopically red coronary thrombi were associated with worse reperfusion and a poorer clinical outcome compared to platelet-rich macroscopically white thrombi (8, 9). Despite its attractive simplicity, qualitative categorisation of thrombi to “white” and “red” is subjective and non-standardised and often not visually feasible as thrombi can present a mixed of “red” and “white” texture (10). Therefore, a quantitative analytical method that is systematic, reproducible and clinically feasible could serve as an important stratification tool for STEACS care during pPCI.

Our group has validated one such approach using reflectance spectroscopy (11). In brief, analysis of the spectrum of visible light reflected from a sample provides information about its molecular composition (12). We have shown that reflectance spectroscopy of the sample can rapidly (near real-time) and reliably identify visually red thrombi and can discriminate between patients with significant and insignificant microvascular injury (11). In this work, we expand our earlier work by spatially resolving the reflectance spectral analysis and using the well-known classification technique of *k*-means clustering (13) in order to automatically quantify the aspirated thrombus and characterise its composition on a pixel by pixel basis. We also explore the potential risk stratification role of reflectance spectroscopy by correlating the thrombus spectra with established invasive and non-invasive indices of coronary microvascular injury.

## Materials and methods

**FIGURE 1:**
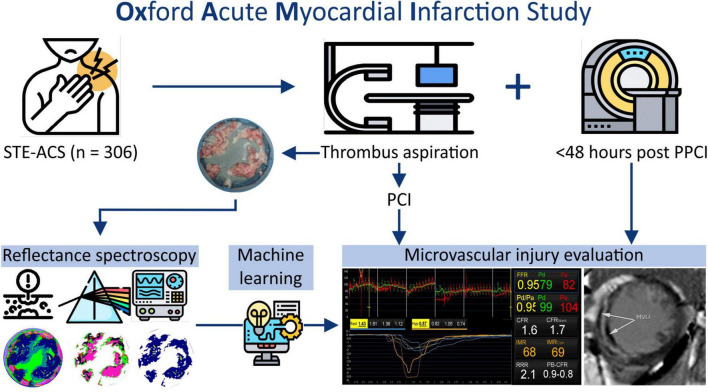
Experimental design.

Patients presenting with a STEMI between June 2012 and December 2020 at the Oxford Heart Centre were recruited in the prospective OxAMI (Oxford Acute Myocardial Infarction) cohort study (14). The current study includes prospectively enrolled participants who underwent manual thrombectomy during primary percutaneous coronary intervention (pPCI) followed by microvascular injury phenotyping (Figure 1). The detailed study flow diagram is shown in Supplementary Figure 1. The study protocol was approved by the local ethics committee (10/H0408/24) and conducted in accordance with the Declaration of Helsinki.

### Manual thrombectomy

Primary percutaneous coronary intervention was performed in standard fashion with the use of adjunctive manual thrombectomy at the operator’s discretion in participants with high thrombus burden. After flow was established in the culprit artery with a 0.014’ angioplasty wire, manual thrombectomy was performed using a conventional 6 French compatible thrombus aspiration catheter - Export (Medtronic), Vmax (Stron Medical), or Hunter (IHT Cordynamic). The chosen thrombectomy device was advanced proximal to the culprit lesion under fluoroscopic guidance and then manoeuvred gently forward and backward while vacuum-based-suction was applied with a 20 ml Luer-lock syringe connected to the proximal hub of the thrombectomy catheter. The aspirate was filtered using a 40 μm pore cell strainer (BD Falcon, Milan, Italy) and collected thrombotic debris was gently washed with normal saline to remove excess blood. The debris within the filter was frozen at −80°C. For this study, a thrombectomy was considered “successful” when the actual aspirated thrombus was representative of the expected thrombus based on the angiographic thrombus burden (see Section “Angiographic analysis”).

### Angiographic analysis

Intracoronary thrombus burden was angiographically evaluated in five grades after flow restoration as previously described (15). Thrombosis In Myocardial Infarction (TIMI) flow and myocardial blush were assessed as previously reported (16, 17).

### Microvascular injury evaluation

Microvascular injury evaluation in OxAMI is performed by two modalities; invasive coronary physiology at the end of the pPCI and cardiac magnetic resonance imaging within 48 h following pPCI (4).

Invasive assessment of the infarct-related artery was performed with commercially available pressure wire technology (Pressure Wire X, Abbott, CA, United States or Certus, St. Jude Medical, MN, United States) and a thermodilution technique as previously described (Supplementary material). The index of microcirculatory resistance (IMR), a well-described index of microvascular injury in STEMI (14), was computed as:


I⁢M⁢R=h⁢y⁢p⁢e⁢r⁢a⁢e⁢m⁢i⁢c⁢P⁢d⁢(m⁢m⁢H⁢g)⁢x⁢a⁢v⁢e⁢r⁢a⁢g⁢e⁢t⁢r⁢a⁢n⁢s⁢i⁢t⁢t⁢i⁢m⁢e⁢(s)

where, *Pd* is the mean distal coronary pressure.

Based on established literature, *IMR* was dichotomized using the clinically significant threshold of 40 U (18).

Non-invasive evaluation by cardiac magnetic resonance imaging was performed as described previously (4) using a 3.0 Tesla scanner (either MAGNETOM TIM Trio or MAGNETOM Verio, Siemens Healthcare, Erlangen, Germany). Microvascular obstruction and infarct size were evaluated and quantified by late gadolinium enhancement (LGE) (Supplementary material). The quantification of infarct size (IS) as a percentage of left ventricular (LV) mass, was performed by setting the signal intensity threshold at 5 standard deviations (SDs) above the mean signal intensity of the remote reference myocardium (19). Microvascular obstruction was identified as the hypointense area within the LGE region and quantified by manual delineation. MVO is expressed as a percentage of LV mass and was dichotomised using the prognostically significant threshold of 1.55% (20). Image analyses were performed on the Cvi42 image analysis software (Circle Cardiovascular Imaging Inc., Calgary, Canada).

### Quantification of thrombus composition by hyperspectral imaging (Experiment A)

The experimental setup for the hyperspectral imaging measurements is shown in Supplementary Figure 2. The cell strainer containing the frozen thrombus sample was illuminated by four 20 W halogen lamps and the reflected light was imaged with the IMEC Snapscan Hyperspectral imaging camera (IMEC, Belgium). Typically, acquisition can be performed in approximately 1 min. For each pixel in the image, the reflected light intensity was recorded at 150 wavelengths across the range 470–900 nm (see Supplementary material for further detail). Spectral images were also acquired for frozen samples of plasma and red blood cells, and for an empty filter and water ice. These were used as basis spectra in the fitting procedure described below.

Two distinct approaches were used to quantify the composition of the material imaged in each pixel based on the pixel’s reflectance spectrum. In the first method, we used an unsupervised machine learning method known as *k*-means clustering (21, 22) to classify the pixels into a user-defined number of groups. Full details of this approach are provided in the Supplementary material. The outcome of this analysis is a set of characteristic “*k*-fractions” for each hyperspectral image, which quantify the spectral composition of pixels identified as thrombus.

In the second approach, we made the assumption that the spectrum for a given pixel can be written as a linear combination of the basis spectra S_plasma_ (λ), S_RBCs_ (λ), S_filter_ (λ), and S_ice_ (λ) recorded for plasma and red blood cells from healthy volunteer and for the filter base and water ice, respectively. The pixel spectra were fitted to the following expression:


S(λ)thrombus=c+0cSplasma(λ)plasma+cSRBCs(λ)RBCs

(1) +cSfilter(λ)filter+cSice(λ)ice.

where c_plasma_, c_RBCs_, c_filter_, and c_ice_ are the fitting coefficients for each basis spectrum, proportional to the weighting of the relevant component in the measured spectrum, and c_0_ is a constant offset. Within this analysis the composition of each pixel is characterised by the set of five fitting coefficients.

### Correlation between thrombus spectral images and microvascular injury (Experiment B)

The relationship between spectral data (in the form of *k*-fractions *f*_*k*_ for each sample) and microvascular injury indices (IMR and MVO) was modelled by fitting the thrombus pixel data set to a range of linear and non-linear multiple linear regressions models. The four models used were:


(2) y=f⁢i⁢tc+0cf1+1cf2+2…+cfkk

(3) y=f⁢i⁢tcf0f1c1…1c2fkck

(4) y=f⁢i⁢tce0xp{-(cf1+1cf2+2…+cfk)k}

(5) y=f⁢i⁢tc[1-exp{-(cf1+1cf2+2…+cfk)k}]0

where *y*_*fit*_ is the fitted value of MVO or IMR, *f*_*k*_ are the *k*-fractions extracted from the spectral image of the patient’s thrombus sample, and *c*_*k*_ are fitting coefficients.

Initially, the above analysis was performed using data from all patients for whom thrombectomy had been attempted (see Supplementary Figure 3). However, it soon became apparent that in many cases sub-optimal thrombectomy was achieved such that the collected thrombus was only a small fraction of the total *in situ* coronary thrombus. The presence of these unrepresentative samples within the data set tends to mask the correlations under study to a significant degree. To address this, we developed a thresholding method (described in detail in the Supplementary material) to identify samples from patients for whom thrombectomy had been “successful,” and repeated the analysis using only these patient samples (*n* = 36).

### Statistical analysis

Following normality assumption evaluation, variables were expressed either as mean ± standard deviation or median (25th to 75th percentile) and categorical variables as numbers (percentage). Multiple linear regression analyses were performed to model the relationship between spectral data and microvascular injury indices. Goodness of fit was evaluated using the co-efficient of determination (*R*^2^). An *R*^2^ value above 0.2 was considered biologically notable. All *p*-values are two-sided whilst *p* < 0.05 was considered statistically significant. All analyses were conducted in MATLAB 2020a (23).

## Results

**FIGURE 2:**
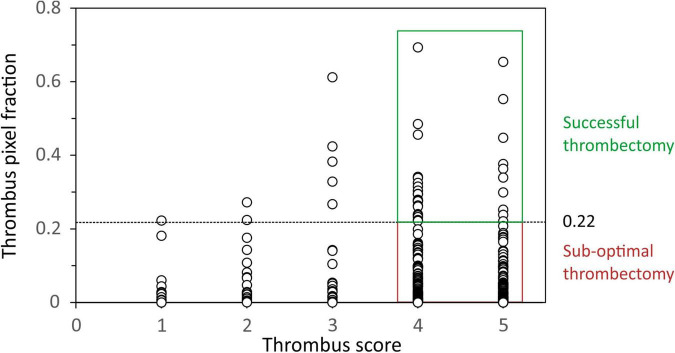
Plot of the thrombus area within each spectral image (expressed as a fraction) against Thrombosis In Myocardial Infarction (TIMI) thrombus score. A threshold thrombus area fraction of 0.22 was used as the minimum value to define successful thrombectomy in patients with thrombus scores of 4 and 5.

In experiment A, a total of 306 patients underwent manual thrombectomy during pPCI yielding a cohort with clinical and procedural characteristics representative of contemporary STEMI patients (Table 1). In experiment B, a cohort of *n* = 36 patients had microvascular injury evaluation and thrombus aspirate samples representative of the angiographic thrombus burden (Figure 2). The clinical and procedural characteristics were comparable to the wider cohort (Table 1).

**TABLE 1:** Clinical, procedural, and coronary microvascular injury characteristics.

	Experiment A	Experiment B
**Total number**	**306**	**36**
**Clinical**		
Age, years	62 **±** 12	61 **±** 11
Male gender, *n* (%)	251 (82)	28 (78)
Hypertension, *n* (%)	130 (42)	14 (39)
Hypercholesterolemia, *n* (%)	118 (39)	17 (49)
Diabetes, *n* (%)	43 (14)	4 (11)
Smoker, *n* (%)	211 (70)	31 (86)
Previous cardiology history, *n* (%)	54 (18)	3 (8)
Family history of IHD, *n* (%)	123 (40)	16 (44)
**Procedural**		
Ischemic time, minutes	262 (122, 273)	202 (101, 234)
Late presenter 6 h, *n* (%)	52 (17)	3 (8)
*Culprit vessel*		
LAD, *n* (%)	138 (45)	15 (42)
LCX, *n* (%)	31 (10)	1 (3)
RCA, *n* (%)	136 (44)	20 (55)
Angiographic thombus score > 3, *n* (%)	238 (82)	27 (85)
TIMI flow–pre-PCI, *n* (%)		
0	237 (78)	28 (78)
1	28 (9)	7 (19)
2	24 (8)	0 (0)
3	15 (5)	1 (3)
TIMI flow–post-PCI, *n* (%)		
0	0 (0)	0 (0)
1	3 (1)	0 (0)
2	44 (14)	2 (6)
3	258 (85)	34 (94)
Myocardial blush grade, *n* (%)		
0	12 (4)	3 (9)
1	28 (10)	3 (9)
2	164 (56)	17 (52)
3	86 (30)	10 (30)
GpIIb/IIIa inhibitor use, *n* (%)	39 (13)	8 (22)
**Complete ST segment resolution, *n* (%)**	219 (74)	28 (74)
**Coronary microvascular injury**		
IMR (U)	50 (22, 69)	49 (19, 61)
IMR > 40 U, *n* (%)	77 (40)	8 (36)
MVO (%)	3 (0, 4)	2 (0,4)
MVO > 1.55%, *n* (%)	63 (39)	8 (44)
Severe CMD (IMR > 40U and MVO), *n* (%)	22 (22)	2 (25)

CMD, coronary microvascular dysfunction; GPIIbIIIa, glycoprotein IIbIIIa; IHD, ischaemic heart disease; IMR, index of microcirculatory resistance; IQR, interquartile range; LAD, left anterior descending; LCx, left circumflex; MVO, microvascular obstruction; PCI, percutaneous coronary intervention; RCA, right coronary artery; TIMI, thrombolysis in myocardial infarction.

### Quantification of thrombus composition by hyperspectral imaging (Experiment A)

**FIGURE 3:**
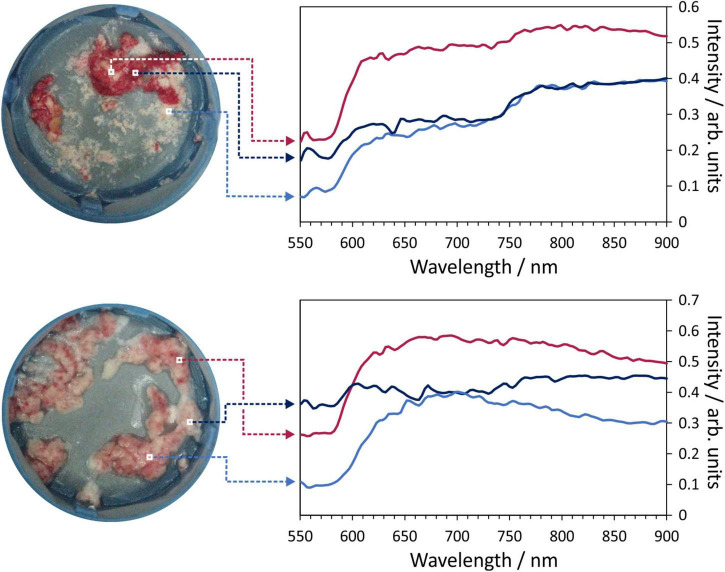
Example hyperspectral images for two thrombus samples. Each image pixel contains a 150-wavelength reflectance spectrum which characterises the composition of the material being imaged. Example spectra for the marked pixels are shown on the right of the figure.

Figure 3 shows examples of the hyperspectral images obtained for two selected thrombus samples. Within the image data, we have information on a spectrum of 150 wavelengths within each pixel. Colour images of the samples are shown on left, with spectra for a few selected pixels within each image shown to the right of the images.

**FIGURE 4:**
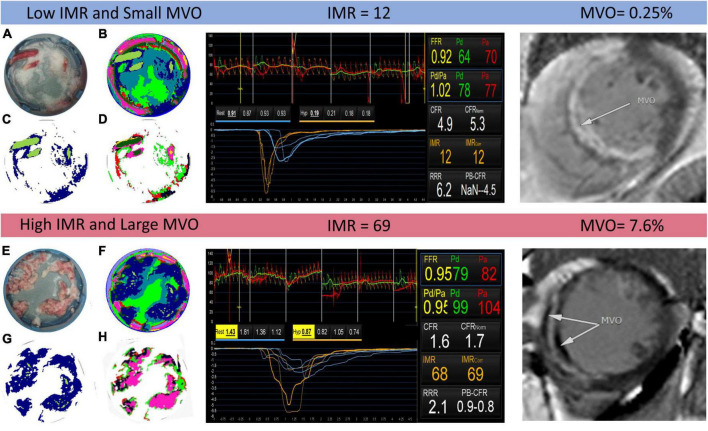
Example output of the *k*-means clustering analysis for two patients identified as having low and high index of coronary microvascular resistance (IMR) (centre panel) and small and large microvascular obstruction (MVO) (right panel), respectively. In the left panel, images **(A,E)** are photographs of the thrombus samples for each patient; images **(B,F)** show the results of the *K* = 11 *k*-means clustering analysis, with each colour corresponding to a different cluster; images **(C,G)** are the same as images **(B,F)** but with all non-thrombus pixels set to zero; and images **(D,H)** show the results of a second *k*-means clustering analysis with *K* = 7, performed only on thrombus pixels.

The *k*-means analysis described in Section “Quantification of thrombus composition by hyperspectral imaging (Experiment A)” and in the Supplementary material was successfully employed across the full data set of sample spectral images, with false colour images generated using the pixel groups assigned by the *k*-means clustering algorithm showing good correspondence with conventional photographs of each sample. Two examples are shown in Figure 4, for two patients identified as having low and high IMR and MVO values, respectively. The left panel of the figure shows photographs of the thrombus samples for the two patients (Figures 4A,E), false colour images showing the pixels assigned to each of the 11 *k*-groups (Figures 4B,F), the same images with all non-thrombus pixels set to zero (Figures 4C,G), and finally, the results of the second *k*-means analysis with *K* = 7 (i.e., seven *k*-groups) carried out only on the thrombus pixels (Figures 4D,H). Note that the choice of 11 and 7 groups for the spectra is explained in the Supplementary material. The central panel of Figure 4 shows screenshots from invasive coronary physiology measurements and the right-hand panel shows the cardiac MRI scans used to determine MVO for the two patients.

We note that when using *K* = 11 in the initial *k*-means analysis on the complete data set, the algorithm, the clustering algorithm was successful in differentiating between the various materials present within the sample, including distinguishing blood and thrombus in blood-contaminated samples. The clusters with *k* = 9 and *k* = 11 were assigned to thrombus and used to generate the images shown in Figures 4C,D. Notably, the spectrum corresponding to the *k* = 11 cluster closely resembles that of the known spectrum of red blood cells, which is dominated by the absorption spectrum of haemoglobin.

**FIGURE 5:**
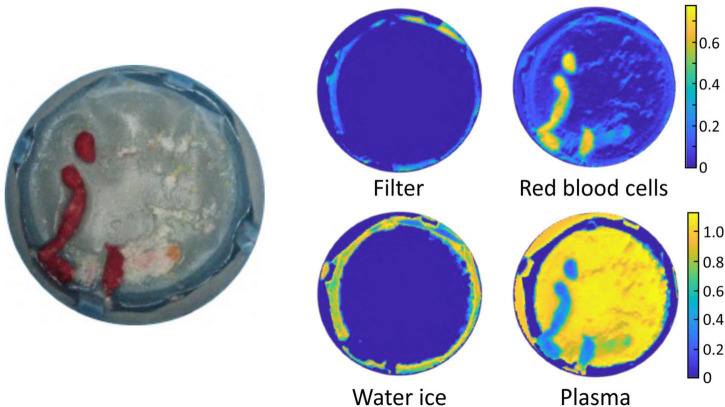
Example output of the basis function fitting process. The image on the left is a photograph of the sample. The false colour images show the fitted contributions to the spectral image from the filter, water ice, plasma, and red blood cells.

As noted in the Section “Materials and methods,” in an alternative analysis the reflectance spectra of plasma, RBCs, empty filter, and water ice were used as basis spectra to perform a linear fit to the spectrum of each image pixel (Eq. 1). Figure 5 shows an example output of the basis function fitting analysis, in which the best-fit coefficients for each spectral component (basis spectrum) are plotted separately as colour maps. Good fits were obtained to Eq. 1, as determined from χ^2^ values and visual inspection, suggesting that these components account for most of the features observed in the reflectance spectra.

### Correlation between thrombi spectra and microvascular injury (Experiment B)

**FIGURE 6:**
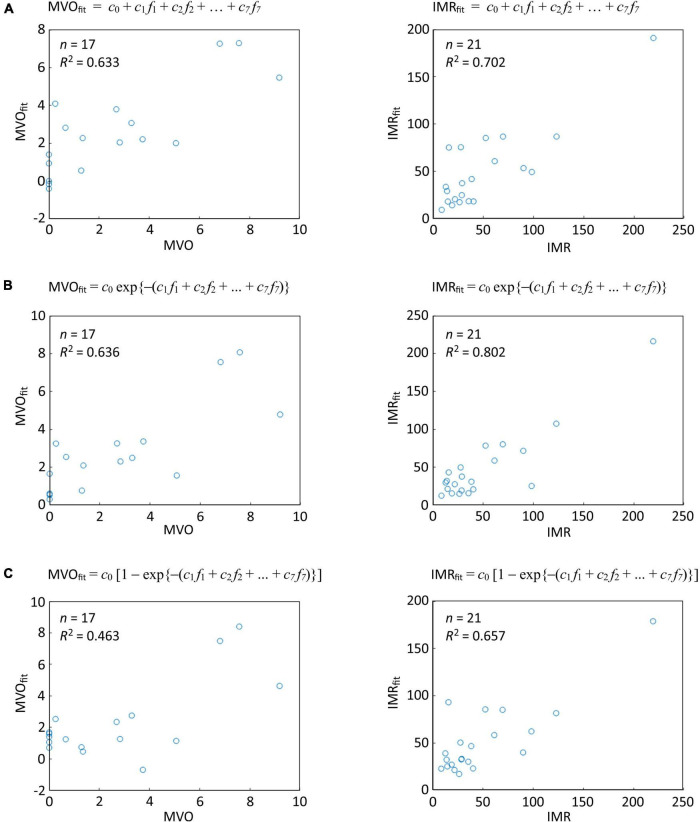
Linear regression analysis of correlations between thrombus spectral parameters predicted microvascular injury and actual microvascular injury indices for the thresholded data set of Oxford Acute Myocardial Infarction (OxAMI) samples. The plots show fits of the thrombus pixel *k*-fractions determined from the spectral images in the thresholded data set to **(A)** Eq. 2; **(B)** Eq. 4; and **(C)** Eq. 5. Note that the fitting coefficients *c*_*k*_ are different for each fit. Sample number *n* and *R*^2^ value are shown for each correlation. Equivalent plots for the full OxAMI data set can be found in Supplementary Figure 3.

Microvascular injury evaluation by CMR and/or IMR was performed in 36 patients, revealing a varied spectrum of microvascular injury in this cohort that was comparable to the injury noted in the wider cohort. As explained in Section “Correlation between thrombus spectral images and microvascular injury (Experiment B),” four different linear regression analyses were performed (Eqs 2–5) in order to model the relationship between the microvascular injury indices and the spectral imaging parameters (*k*-fractions) extracted from the thrombus pixels using *k*-means clustering. The power model (Eq. 3) was not able to fit the data well, and will not be considered further. The results of the analysis using Eqs (2, 4, 5) are shown in Figure 6.

The third model (Eq. 4), which assumes an exponential dependence of IMR and MVO on the *k*-fractions, performed best in predicting IMR (*R*^2^: 0.80, *p* < 0.0001, *n* = 21) and MVO (*R*^2^: 0.64, *p* = 0.02, *n* = 17).

## Discussion

Coronary microvascular dysfunction in the STEACS setting is prognostically important (4, 5, 18), and its early and reliable identification during pPCI can guide stratification of adjunct therapies (24–26). Early work has shown that macroscopically red thrombi are associated with an adverse prognostic outcome (8, 9). Our proof-of-concept work established the feasibility of reflectance spectroscopy as a novel standardised tool for intraprocedural risk stratification. This study expands on our preliminary findings (11) by applying a robust, automated, near real-time analytical technique for coronary thrombus reflectance spectral characterisation and exploring its diagnostic role by correlating it with established coronary microvascular function indices.

In this work we have employed for the first time spatially resolved reflectance spectral analysis of coronary thrombus samples, in which reflectance spectra are recorded for every pixel in the image of each sample. This capability has enabled us to use advanced processing methods to: (i) phenotype the spectral signature of aspirated thrombi through machine learning approaches (unsupervised *k*-means clustering); and (ii) use the results of the clustering analysis to determine the regions of the images corresponding to aspirated thrombus area in an automated fashion. We have shown that separation of the pixel spectra into 11 groups allows the successful assignment of the various image components (thrombus, blood, water ice, filter, etc.).

Having developed a reliable method for determining the amount of aspirated thrombus, we observed that the adjunctive use of manual thrombectomy in patients with high angiographic thrombus burden only rarely led to aspiration of thrombus amounts larger than those aspirated from patients with low angiographic thrombus burden. This suggests that manual thrombectomy is infrequently effective at modifying thrombus burden, corroborating earlier work (27). Indeed, residual thrombus has been associated with worse microvascular dysfunction in STEACS (28), and sub-optimal thrombectomy has been put forth as one of the reasons that thrombus modification in STEACS was clinically inefficacious (7).

Finally, we have shown that the reflectance spectral signatures of the aspirated coronary thrombi show clear and reliable correlation with the degree of microvascular injury, as measured by MVO and IMR. The observed correlations are not perfect, highlighting that thrombus composition is one of the multiple factors influencing the extent of microvascular injury (29). Nonetheless, our work complements and improves on our earlier findings, which showed that spectrally identified red-cell content was able to modestly segregate patients with clinically significant and insignificant microvascular injury following a STEACS (11). Taking these two studies together, our work has shown that reflectance spectroscopy can offer a standardised, rapid, and reliable technique for identifying and stratifying patients with significant microvascular injury in cases where representative amounts of coronary thrombus are available for analysis.

## Limitations

Firstly, we note that while the present work was carried out on frozen samples collected as part of the OxAMI study, the method is easily extendable to fresh samples. Further work is also underway which employs alternative spectroscopic and spectrometric methods with greater discriminating power. Together these methods have the potential to lead to a novel, high throughput, and non-destructive tool to study the pathobiology of coronary thrombus.

It is also worth mentioning that the 8 years over which patients were enrolled in the OxAMI study saw changes in pharmacotherapy that could have influenced aspirated thrombus composition. However, 97% of participants were pre-loaded with DAPT prior to their PPCI and 93% had intraprocedural bivalirudin administered. Adjunctive pharmacotherapy in the form of GpIIb/IIIa was used in 13% of all patients and a comparable 22% in patients from cohort B. Indeed, GpIIb/IIIa was predominantly (72%) used as a bailout strategy after thrombus aspiration. Nonetheless this is unlikely to confound our endpoint as insights from the T-TIME randomised trial show that intracoronary thrombolysis had no effect on microvascular injury indices (30).

Finally, from an external validity perspective, our approach can be applied to the small number of STEACS patients undergoing manual thrombectomy in contemporary practice (31). However, the early phase RETRIEVE-AMI (NCT05307965) and NATURE (NCT04969471) trials exploring novel tools for thrombus retrieval offer an excellent opportunity for our technology, as they address the drawbacks that underpin the dwindling use of aspiration thrombectomy and aim to define and refine its exact role in the expanding landscape of intraprocedural risk stratification tools for STEACS care. While we acknowledge that the correlations between spectral data and clinical parameters observed in the present study are somewhat limited by the small number of patients included in the thresholded data set, we are working to address this by employing alternative approaches involving analysis of plasma from coronary aspirate. These newer approaches do not require thrombectomy and can therefore be generalised to all patients undergoing pPCI.

## Conclusion

We have shown that reflectance spectral imaging of coronary thrombus combined with machine learning approaches enables the determination of parameters correlating with thrombus composition, including automated quantification of thrombus area within the images. We have also shown for the first time that spectral signatures of coronary thrombi correlate with microvascular injury indices in patients with STEACS. Further validation of this point-of-care system in future studies will potentially enable the integration of reflectance spectroscopy into the diagnostic workflow of STEACS and facilitate the stratified deployment of adjunct treatment therapies.

## Data availability statement

The datasets presented in this article are not readily available because a patent based on some of the data/approaches included in this article has been filed. Requests to access the datasets should be directed to GD, GiovanniLuigi.Demaria@ouh.nhs.uk.

## Ethics statement

The studies involving human participants were reviewed and approved by NRES Committee South Central – Oxford C (REC Number: 10/H0408/24). The patients/participants provided their written informed consent to participate in this study.

## Author contributions

RK: methodology, investigation, writing – original draft, review and editing, visualisation, project administration, and funding acquisition. KF: methodology, investigation, formal analysis, visualisation, writing – review and editing, and project administration. CG: formal analysis, visualisation, and writing – review and editing. RL, MA, FM, and ME: investigation and writing – review and editing. AB: investigation, resources, writing – review and editing, and funding acquisition. CV and GD: conceptualisation, methodology, investigation, resources, writing – original draft, review and editing, supervision, project administration, and funding acquisition. KC: conceptualisation, methodology, investigation, resources, writing – review and editing, project administration, and funding acquisition. All authors contributed to the article and approved the submitted version.

## Abbreviations

MR: index of microcirculatory resistance
LV: left ventricular
MVO: microvascular obstruction
pPCI: primary percutaneous coronary intervention
STEACS: ST-segment elevation acute coronary syndrome.
