# Supplementary material for: Machine learning assisted reflectance spectral characterisation of coronary thrombi correlates with microvascular injury in patients with ST-segment elevation acute coronary syndrome
Source: Front Cardiovasc Med. 2022 Sep 20;9:930015. doi: 10.3389/fcvm.2022.930015 (PMC9530633; doi:10.3389/fcvm.2022.930015)
Supplement: Supplementary file 1 [file Data_Sheet_1.docx]

**Supplementary Methods**

**Coronary physiology evaluation**

Evaluations were performed following intracoronary injection of 250μg of isosorbide dinitrate. Average transit time was measured following three discrete 3ml injections of room temperature normal saline at resting and hyperaemic conditions. Hyperaemia was induced by intravenous adenosine infusion at a rate of 140μg/kg/min.

Patients with i) haemodynamic instability, ii) previous coronary artery bypass grafting, iii) severe chronic renal impairment, iv) severe left main angiographic stenosis or complex coronary anatomy (tortuosity, chronic total occlusion) and v) treated with inly plain old balloon angioplasty pPCI were excluded.

**Cardiovascular Magnetic Resonance (CMR) Protocol**

LGE was performed with a T1-weighted segmented inversion recovery gradient echo-phase sensitive-inversion recovery (GRE_PSIR) sequence (TE/TR = 2.5 msec/5 msec, voxel size =1.8 x 1.4 x 8.0 mm, flip angle 20°). LGE images were collected 10-15 min after the administration of 0.1 mmol/kg contrast agent (Dotarem, Guerbet, Villepinte, France). The inversion time was adjusted for optimal nulling of remote normal myocardium.

**Hyperspectral image processing**

Within the IMEC HSImager acquisition software, the spectral images were divided by the spectral image recorded for a white reflectance standard in order to correct for variations in the intensity of the light source at different wavelengths across the range of interest. Before being subjected to the data analysis procedures described in the following, each data set was cropped to the wavelength range of 550-900 nm to remove a region of poor signal-to-noise at short wavelengths caused by low intensity of the light source. The final data set for each thrombus sample comprised a 250 x 250 x 112 hypercube with two spatial dimensions (250 x 250 pixels) and one wavelength dimension (112 wavelengths).

**Quantifying thrombus composition by unsupervised machine learning**

We used an unsupervised machine learning method known as *k*-means clustering(1, 2) to classify the pixels into a user-defined number of groups. Each spectrum is treated as a vector in 112 dimensional space, and the algorithm identifies the optimum groupings of spectra in an iterative process based on minimizing the distances between the vectors assigned to each group. Clustering was performed across all pixels from the entire data set of spectral images. The composition of each spectral image (i.e. each sample) was then quantified by a set of ‘*k*-fractions’, defined as the number of pixels classified into each *k* group divided by the total number of pixels in the image. Before calculating the *k*-fractions the groups were reordered such that *k* = 1 corresponded to the most populated group (across the entire data set), *k* = 2 to the next most populated group, and so on. In order to correlate the *k*-fractions with clinical data (see Section 2.5), it makes sense to focus only on pixels identified as thrombus within the iages. To achieve this, the *k*-clusters corresponding to thrombus were identified from a *k*-means analysis with *K*=11, and the reduced data set comprising only thrombus pixels was subjected to a second *k*-means clustering analysis with *K* = 7. The *k*-fractions determined in this second analysis were then correlated with clinical parameters as described in Section 2.5. The optimum number of *k*-groups to use in each analysis was determined using an elbow plot(3-6), which quantifies the balance between including enough groups to describe the main features of the data set, but not so many groups as to risk overfitting the data set.

**Defining a “successful” thrombectomy via a thresholding method**

The thresholding process was based on the hypothesis that the volume of thrombus extracted from patients assigned as having a high thrombus burden (defined as an angiographic thrombus score >3) is expected to be higher than the maximum thrombus volume retrieved in patients with a low thrombus burden (angiographic thrombus score 3). For each patient, the number of pixels in the corresponding spectral image that were assigned to thrombus, quantified through the corresponding *k*-fractions (see section above) was used as a proxy for thrombus volume. A plot of the sum of the thrombus pixel fraction against the TIMI thrombus score is shown in Figure 2. While there are some outliers, the data for the low thrombus-score patients shows that the vast majority have a thrombus *k*-fraction <0.22, leading us to choose this value as the threshold for including patients with higher thrombus scores in the analysis, i.e. patients with a TIMI thrombus score of 3 or 4 were deemed to have had a “successful” thrombectomy if their thrombus pixel *k*-fraction was greater than 0.22.

**Supplementary Figures**

**
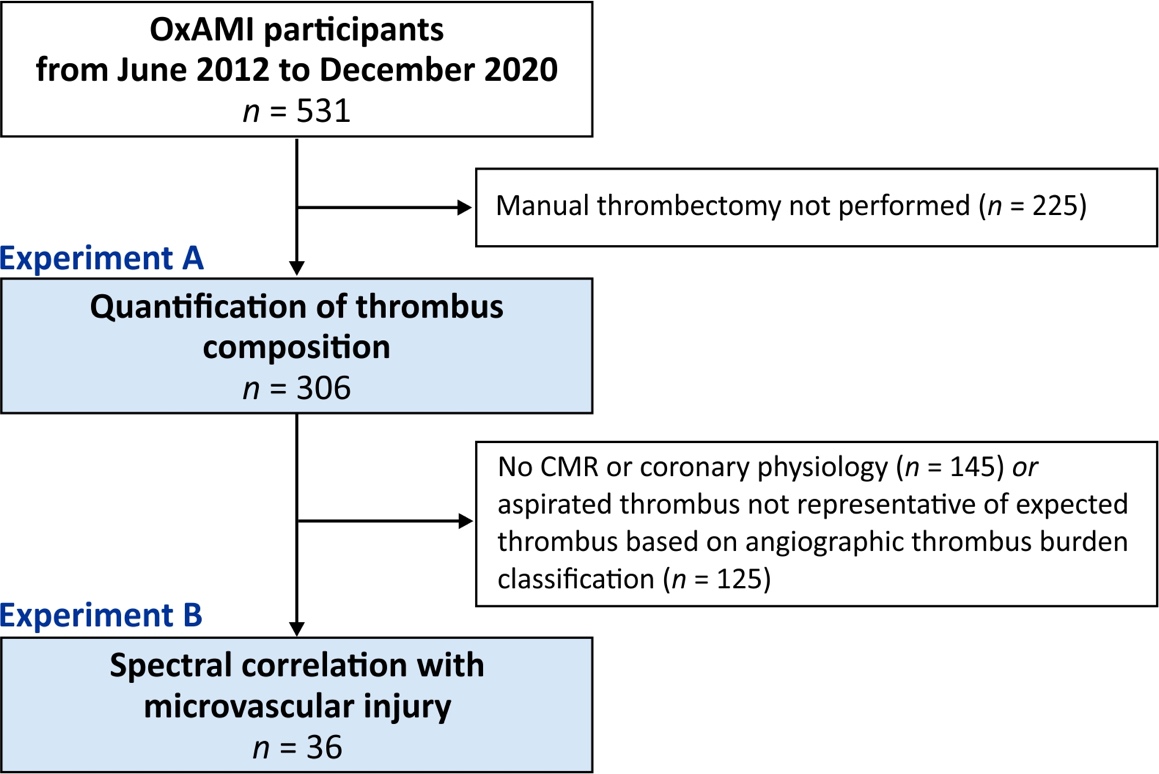
**

**Supplementary Figure 1:** Study flow diagram. CMR = cardiac magnetic resonance.

**
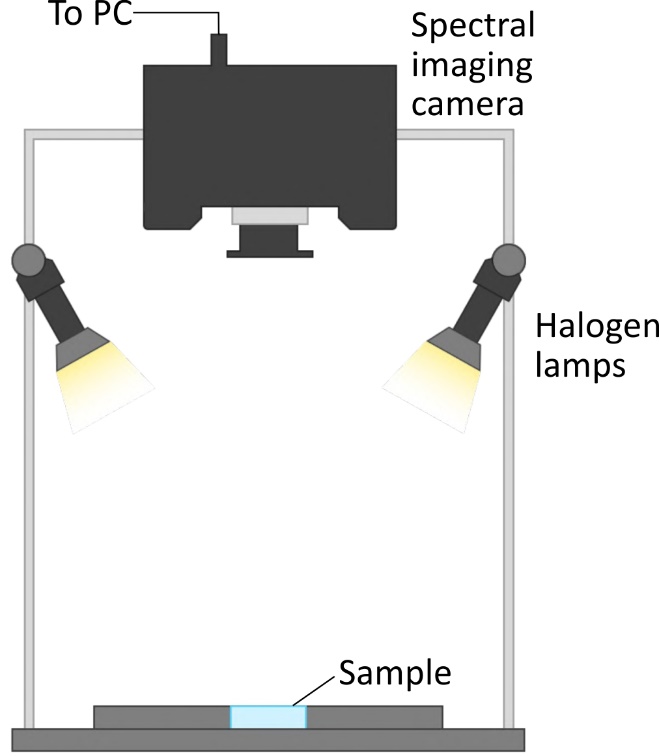
**

**Supplementary Figure 2:** Schematic of the experimental setup for the reflectance spectral imaging measurements.

**
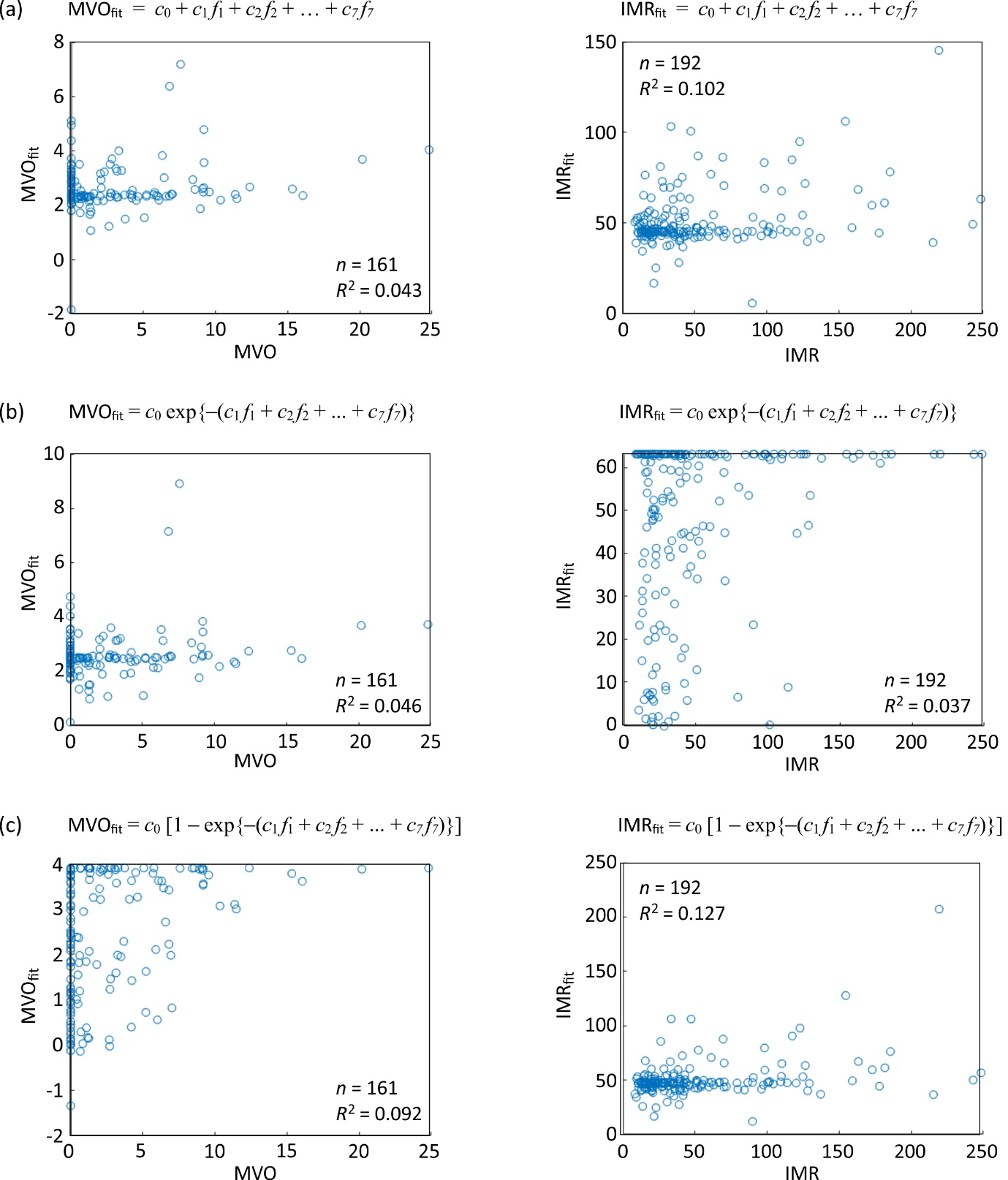
**

**Supplementary Figure 3**: Linear regression analysis of correlations between thrombus spectral parameters predicted microvascular injury and actual microvascular injury indices for the complete data set of OxAMI samples before thresholding. The plots show fits of the thrombus pixel *k*-fractions determined from the spectral images in the thresholded data set to (a) Equation (2); (b) Equation (4); and (c) Equation (5). Note that the fitting coefficients *c_k_* are different for each fit. Sample number *n* and *R*^2^ value are shown for each correlation. These results should be compared with those shown in Figure 6 of the manuscript, in which only samples from patients determined to have had a successful thrombectomy are included in the analysis. The correlations are much improved in the thresholded data set.

**Supplementary References**

1. Forgy EW. Cluster analysis of multivariate data : efficiency versus interpretability of classifications. Biometrics. 1965;21:768-9.

2. Lloyd S. Least squares quantization in PCM. IEEE Transactions on Information Theory. 1982;28(2):129-37.

3. Bholowalia P, Kumar A. EBK-Means: A Clustering Technique based on Elbow Method and K-Means in WSN. International Journal of Computer Applications. 2014;105:17-24.

4. Kodinariya TM, Makwana PR, editors. Review on determining number of Cluster in K-Means Clustering2013.

5. Liu L, Peng Z, Wu H, Jiao H, Yu Y, Zhao J. Fast Identification of Urban Sprawl Based on K-Means Clustering with Population Density and Local Spatial Entropy. Sustainability. 2018;10(8):2683.

6. Lopez-Rubio E, Palomo EJ, Ortega-Zamorano F. Unsupervised learning by cluster quality optimization. Information Sciences. 2018;436:31-55.
